# A novel knockout mouse for the small EDRK-rich factor 2 (*Serf2*) showing developmental and other deficits

**DOI:** 10.1007/s00335-021-09864-6

**Published:** 2021-03-13

**Authors:** Karen Cleverley, Weaverly Colleen Lee, Paige Mumford, Toby Collins, Matthew Rickman, Thomas J. Cunningham, James Cleak, Joffrey Mianne, Zsombor Szoke-Kovacs, Michelle Stewart, Lydia Teboul, Cheryl Maduro, Sara Wells, Frances K. Wiseman, Elizabeth M. C. Fisher

**Affiliations:** 1 Department of Neuromuscular Diseases, Queen Square Institute of Neurology, London, UK; 2 The UK Dementia Research Institute, University College London, Queen Square, London, WC1N 3BG UK; 3 Mammalian Genetics Unit, Harwell, UK; 4 Mary Lyon Centre, MRC Harwell Institute, Oxfordshire, OX11 0RD UK

## Abstract

The small EDRK-rich factor 2 (SERF2) is a highly conserved protein that modifies amyloid fibre assembly in vitro and promotes protein misfolding. However, the role of SERF2 in regulating age-related proteotoxicity remains largely unexplored due to a lack of in vivo models. Here, we report the generation of *Serf2* knockout mice using an ES cell targeting approach, with *Serf2* knockout alleles being bred onto different defined genetic backgrounds. We highlight phenotyping data from heterozygous *Serf2*^+/−^ mice, including unexpected male-specific phenotypes in startle response and pre-pulse inhibition. We report embryonic lethality in *Serf2*^−/−^ null animals when bred onto a C57BL/6 N background. However, homozygous null animals were viable on a mixed genetic background and, remarkably, developed without obvious abnormalities. The *Serf2* knockout mice provide a powerful tool to further investigate the role of SERF2 protein in previously unexplored pathophysiological pathways in the context of a whole organism.

**Supplementary Information:**

The online version contains supplementary material available at 10.1007/s00335-021-09864-6.

## Introduction

The molecular events that occur in cells during ageing can inform our understanding of age-related diseases. Many neurodegenerative diseases share the hallmark appearance of fibrillar protein aggregates in the brain, and while the role of these aggregates in disease remains unclear, such fibrillary structures are composed of aggregation-prone proteins, for example, mutant huntingtin (HTT) in Huntington disease, α-synuclein in Parkinson disease and amyloid-beta (Aβ) in Alzheimer disease (Scherzinger et al. 1999; Chiti and Dobson 2006; Goedert and Spillantini 2006). Although the contribution of aberrantly folded protein aggregates to the pathogenesis of disease is not fully understood, the current most prevalent hypothesis is that aggregation intermediates are toxic to cells and that the aggregation process is a cellular protection mechanism against these cytotoxic intermediates (Lansbury and Lashuel 2006; Hartl and Hayer-Hartl 2009; Ogen-Shtern, Ben David, and Lederkremer 2016).

SERF proteins are evolutionarily conserved and positively regulate protein aggregate formation (van Ham et al. 2010; Falsone et al. 2012; Meinen et al. 2019; Merle et al. 2019; Meyer et al. 2020). Genetic screens for modifiers of aggregation in *Caenorhabditis elegans* models led to the identification of Modifier of Aggregation 4 (MOAG-4) (van Ham et al. 2010) and Cytotoxicity-Related Aggregation Mediator-1 (CRAM-1) (Balasubramaniam et al. 2018), the human orthologs of which are SERF1A and SERF2 respectively. These proteins are thought to regulate age-related proteotoxicity through a previously unexplored pathway (Stroo et al. 2017). Inactivation of MOAG-4 suppresses protein aggregation and associated toxicity in *C. elegans* models (van Ham et al. 2010) and SERF1A accelerates the aggregation of amyloidogenic proteins in vitro (Falsone et al. 2012). MOAG-4 also promotes the aggregation of α-synuclein by interfering with intramolecular interactions (Yoshimura et al. 2017). CRAM-1 blocks proteasomal degradation of ubiquitinated proteins and thus promotes aggregation (Ayyadevara et al. 2015) and SERF2 promotes aggregation of Aβ in human neuroblastoma cells overexpressing APP (amyloid precursor protein) (Balasubramaniam et al. 2018).

Native mass spectrometry shows that SERF proteins exhibit a high degree of plasticity and can form fuzzy, highly extended complexes with amyloid-prone proteins such as Aβ40 and α-synuclein (Meinen et al. 2019); this protein–protein interaction is biologically active but disordered, yet is sufficient to potentiate cytotoxic aggregation (Merle et al. 2019). The disordered nature of SERF proteins allows conformational adaptation; SERF1A has been shown to bind RNA, accumulating in the nucleus under physiological conditions; however, under stress conditions SERF1A is rapidly released into the cytosol where it favours binding to α-synuclein (Meyer et al. 2020). This suggests a physiological role and, in stress, a pathological gain-of-interaction for SERF1A. The function of its paralogue, SERF2 also a largely disordered protein, remains to be determined (Balasubramaniam et al. 2018).

Here we describe *Serf2* knockout mice that will provide a platform for the exploration of the role of SERF2 in the aggregation of proteins involved in the pathology of neurodegenerative diseases. Our characterisation includes finding a tissue-specific pattern of expression, a role in development that is essential on one genetic background, and a male-specific phenotype affecting startle response and pre-pulse inhibition in heterozygous knockout animals.

## Materials and methods

### Animal welfare and husbandry

All animals were housed and maintained in the Mary Lyon Centre, MRC Harwell Institute and at UCL, under specific pathogen-free (SPF) conditions, in individually ventilated cages adhering to environmental conditions as outlined in the Home Office Code of Practice. All animal studies were lisensed by the Home Office under the Animals (Scientific Procedures) Act 1986 Amendment Regulations 2012 (SI 4 2012/3039), UK, and additionally approved by the Institutional Ethical Review Committees. All mice were co-housed throughout the study as lone housing is known to modify phenotypes; mice were housed with littermates and/or animals of the same sex weaned at the same time, thus mice of differing genotypes were co-housed pseudorandomly. Mice had access to a mouse house with bedding material and wood chips. All animals had continual access to water and RM1 (Special Diet Services) (stock animals) or RM3 (Special Diet Services) (breeding animals) chow. Animals were euthanized following Schedule 1 methods in accordance with the Animals (Scientific Procedures) Act 1986 (United Kingdom).

### Generation of *Serf2* knockout C57BL/6 mice

**Fig. 1:**
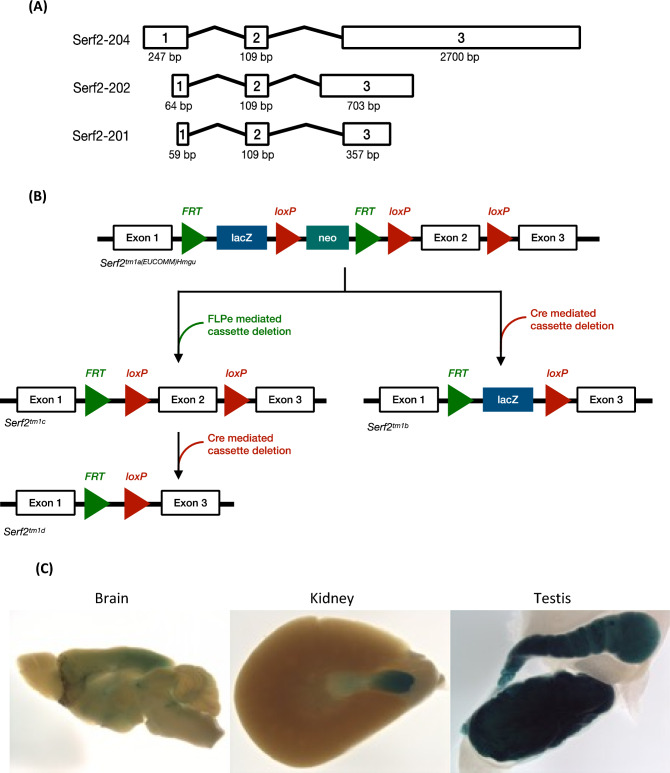
*Serf2*^*tm1b*^ allele and gene expression. **a** Schematic of *Serf2* locus showing alternatively spliced protein coding transcripts published in the mouse Ensembl genome database (Mouse reference assembly GRCm38.p6). **b** International Mouse Knockout Project strategy to create the *Serf2*^*tm1b*^ and *Serf2*^*tm1d*^ alleles. Note: *Serf2*^*tm1a*^ MGI: 5,771,993; *Serf2*^*tm1b*^ MGI: 6,120,704; *Serf2*^*tm1d*^ MGI: 6,317,359. **c** Examples of X-gal staining in 15-week-old heterozygous adults (C57BL/6 N background). Shown from left to right: sagittal brain section, kidney and testis. All organs were a similar size to those of wildtype controls at this age

*Serf2* conditional ready ES cells and mice (allele name: *Serf2*^*tm1a(EUCOMM)Hmgu)*^, MGI:5,771,993) were generated in collaboration with the International Mouse Knockout Consortium (https://www.mousephenotype.org/), via a mouse embryonic stem (ES) cell targeting approach using C57BL/6Ntac ES cells, such that exon 2, a critical exon, was flanked by *loxP* sites (*Serf2* has three exons) (Fig. 1).

Subsequently the Mary Lyon Centre at MRC Harwell used recombination at *loxP* sites via soluble Cre protein (TAT-Cre (Tat-NLS-Cre, HTNC, HTNCre), Excellagen, Rockville, USA) during in vitro fertilisation, to remove critical exon 2 and generate the null *Serf2*^*tm1b*^ allele (MGI: 6,120,704) that expresses a β-gal reporter under the endogenous promotor. An additional IVF was carried out using *Serf2*^*tm1a*^ sperm with oocytes from a Flp recombinase expressing line (Gt(ROSA)26Sor ^tm2(CAG−flpo,−EYFP)Ics^), after fertilisation, embryos at a two-cell stage were exposed to soluble Cre as above, to create the null *Serf2*^*tm1d*^ allele (MGI: 6,317,359) as shown (Fig. 1).

### Animal cohorts

Cohort A: (E14.5 for microCT) WT *n* = 8, *Serf2*^+/−^ *n* = 11, *Serf2*^*−/−*^* n* = 6; tm1b allele, co-isogenic on C57BL/6NTac.

Cohort B: (3-month-old males for protein and qPCR) WT *n* = 4, *Serf2*^+/−^ *n* = 4, *Serf2*^*−/−*^* n* = 3; tm1d allele, mixed background (produced by the *Serf2*^*tm1d*^ allele in multigeneration inter-crosses of 128S8, C57BL/6Nimr and C57BL/6 J).

Cohort C: (3-month-old males and females for behavioural profiling) WT *n* = 2,172, *Serf2*^+/−^ *n* = 15; tm1b allele, co-isogenic on C57BL/6NTac. We note that wildtype animals are the baseline control mice from the International Mouse Phenotyping Consortium pipeline, hence the large number of controls; please see text below.

In addition, 15-week-old males and females of this genotype (tm1b allele)/genetic background (C57BL/6NTac), wildtype and heterozygous littermates, were used for gene expression analysis by X-gal staining.

### DNA extraction and genotyping

DNA was extracted from tail tip or ear biopsy by the Hot Shot method (Truett et al. 2000). Mice were genotyped using polymerase chain reaction (PCR) for the presence of *Serf2* (control primers f: 5′- CTC CGG CGT CTC ACT TTG TAC CT -3′ r: 5′- CAC TCT GCC CCT CAC ATC TAA CC-3′, *Serf2* knockout specific primers f: 5′- AAG GCG CAT AAC GAT ACC AC -3′ r: 5′- ACT GAT GGC GAG CTC AGA CC -3′); giving a 274 bp product for the wildtype (WT) allele and a 174 bp product for the targeted allele. The PCR buffer was: 1 × MegaMix Blue Buffer (supplied as 10 × stock, Microzone) and 1 μM of each genotyping primer, PCR conditions were as follows: 95 °C for 1 min, 30 cycles at 95 °C for 10 s, 60 °C for 10 s, 72 °C for 1 s, and 1 cycle of 72 °C for 30 s. PCR products were resolved by electrophoresis on 2% agarose gels in TBE (Tris–Borate-EDTA) with 1 μg/ml GelRed^®^ (Biotium), run at 100 V and constant current.

### RNA extraction and quantitative RT-PCR

Total cortical RNA was extracted using the RNeasy^®^ mini kit (Qiagen). Tissue (Cohort B animals) was disrupted using a TissueRuptor^®^ (Qiagen), and the protocol followed as per the manufacturer’s instructions, samples were defrosted and homogenised on ice. Final extracted RNA was eluted in DNase- and RNase-free water. Amounts of RNA were equalised and cDNA was generated using the SuperScript™ First-Strand Synthesis System for RT-PCR (Invitrogen). Quantitative PCR was undertaken to determine expression of all isoforms of *Serf2* (primers f: 5′-ATG ACC CGC GGT AAC CAG-3′ r: 5′-GAA GAA GCA GAG CGA CTC GG-3′ probe FAM-CGA GAG CTC GCC CGC CAG AAG AAC A). Mouse β-actin (*Actb*) control mix (4354315 Applied Biosystems) was used as an endogenous control with a VIC^®^ dye-labelled TaqMan^®^ MGB probe. Minus reverse-transcriptase controls were run for every sample for all reactions. No evidence of genomic amplification was detected (Wiseman et al. 2018).

### Tissue preparation and western blotting

For analysis of protein abundance in the cortex, tissue (Cohort B animals) was dissected under ice-cold PBS before snap freezing. Samples were then homogenised in RIPA Buffer (150 mM sodium chloride, 50 mM Tris, 1% NP-40, 0.5% sodium deoxycholate, 0.1% sodium dodecyl sulphate) plus complete protease inhibitors (Calbiochem) by mechanical disruption. Total protein content was determined by Bradford assay. Samples from individual animals were run separately and were not pooled. Equal amounts of total brain proteins were then denatured in LDS denaturing buffer (Invitrogen) and β-mercaptoethanol, prior to separation by SDS-PAGE gel electrophoresis using precast 4–12% Bis–Tris gels (Invitrogen). Proteins were transferred to nitrocellulose membranes prior to blocking in 5% milk/PBST (0.05% Tween-20). Primary antibodies were diluted in 1% BSA/PBST, HRP conjugated secondary anti-rabbit and anti-mouse antibodies (Dako) were diluted 1:5000 in 1% BSA/PBST. Primary antibodies against SERF2 (Proteintech 11691-1-AP, 1:1000) and β-actin (Sigma A5441, 1:60,000) were used.

### Gene expression analysis in adult mice

For analysis of gene expression by X-gal staining (Cohort C) the following protocol was undertaken. Day 1—All solutions at pH 8.0. Adult, anaesthetised mice between 8–16 weeks of age were perfused using ~ 40 ml 4% PFA in PBS over 15 min. A standard set of tissues were extracted (Supplementary Table S1) and placed into 200 ml 4% PFA to fix for a further 30 min on ice. The brain was cut sagittally using a brain matrix into ~ 1 mm sections that were placed into a histological cassette. All tissues were then subject to 3 × 20 min washes in 200 ml 1 × PBS at 4 °C. Tissues were then incubated in 200 ml LacZ solution (2 mM MgCl_2_.H_2_0, 0.02% IGEPAL, 5 mM potassium ferrocyanide, 5 mM potassium ferricyanide, 10% sodium deoxycholate, 1 mg/ml X-gal in dimethylformamide made up in 1 × PBS and filtered using a 0.2 μM steritop filter) for 48 h at 4 °C. Day 3—Tissues were washed 2 × 30 min 1 × PBS followed by an overnight post-fix in 4% PFA. Day 4—Tissues were rinsed with 1 × PBS and kept overnight in 50% glycerol (in PBS). Day 5—The glycerol was replaced with 70% glycerol (in PBS) and samples were kept in the dark at 4 °C until imaging. Imaging was carried out on a Leica M165C stereo microscope using a Jenoptik ProgRes speed XT^core^5 camera and images stored in an iMagic IMS client as a JPEG.

### Sample preparation for MicroCT

Mice were mated and detection of a vaginal plug the following morning was considered to be 0.5 dpc. At 14.5 dpc pregnant females were sacrificed by cervical dislocation and the uterine horns dissected out. Individual embryos (Cohort A animals) were removed from placentae and allowed to bleed out in cold PBS for ~ 10 min. During this period any scab that had formed over the umbilical area was removed to allow for continued bleeding. Embryos were placed in individual wells of a Corning Costar 6-well plate and fixed in 10 ml of 4% PFA, pH 8 on a rocker at 4 °C overnight. Once fixation was confirmed embryos were stored in 1% PFA, pH 8 until ready for potassium tri-iodide (Lugol) staining. Embryos were placed in individual glass bijou bottles with 15 ml of contrast agent, 50% Lugol solution (32922, Sigma-Aldrich) made up in distilled water (dH_2_O). These were wrapped in foil to protect from light and placed on a rocker at room temperature for two days. Following contrast samples were rinsed and then washed with dH_2_O for at least one hour to remove any excess contrast solution. Samples were then embedded in 1% Iberose high specification agarose (AGR-500, Web Scientific) in 4.5 ml CryoTube vials. Samples were left to set in the agarose for a minimum of two hours at room temperature prior to scan initiation.

### MicroCT imaging

For each embryo 3D datasets were acquired using a Skyscan 1172 high resolution microCT scanner (Bruker). Scans were carried out with the X-ray source at 80 kVp and 124 µA and using an aluminium filter. Using the NRecon software supplied with the microCT scanner slices were reconstructed into digital cross-sections by a Feldkamp algorithm for cone beam CT (Feldkamp et al. 1984). The resulting 3D dataset is 4000 × 4000 × 2000 voxels of 2.96 µm.

### MicroCT post-processing

Following reconstruction 3D datasets underwent further processing through an in-house programme, HARP (Brown et al. 2018), which automatically crops tightly around the sample to remove empty data. It also allows for the dataset to be resampled to more manageable sizes. Samples were routinely cropped and resized to a voxel size of 14 µm using HARP. Once post-processed datasets were viewed in 3D Slicer (http://slicer.org/) or FIJI (http://fiji.sc/wiki/index.php/Fiji).

### Acoustic startle and pre-pulse inhibition

Mice from Cohort C were placed in an acoustic startle chamber (Med Associates Inc, USA) and acclimatised to a background noise level of 53 dB for 5 min, followed by exposure to 5 120 dB startle tones. Mice were then exposed to a startle tone at 120 dB for 40 ms, either on its own or preceded 80 ms earlier by a 20 ms pre-pulse at 56, 58 or 65 dB (PPI1, PPI2, PPI3). Responses to the startle tone were measured for 100 ms following the start of the startle tone using a piezoelectric transducer in the floor of the chamber which detected movement of the animal. Each trial condition was tested 10 times. More details available at IMPRESS (www.mousephenotype.org/impress).

## Results

### Generation of Serf2 knockout mice, gene expression pattern in adults, and homozygous early embryonic lethality on a C57BL/6 N background

*Serf2* knockout mice were generated in collaboration with the International Mouse Knockout Project using a mouse embryonic stem (ES) cell targeting approach. The knockout allele, *Serf2*^*tm1b*^ has exon 2 deleted from this three-exon gene (Fig. 1a), and instead expresses a LacZ sequence that is driven by the endogenous promotor and so, after beta-galactosidase staining, indicates spatial and temporal expression of *Serf2* (Fig. 1b) (Skarnes et al. 2011) (Murray 2020).

SERF2 expression in adults (15 weeks of age) was assessed using beta-galactosidase staining from the *Serf2*^*tm1b*^ allele in heterozygous mice on the congenic on C57BL/6Ntac background. Expression was clearly seen in discrete regions within heart, blood vessels, throughout the brain and spinal cord, adrenal gland, kidney, male and female gonads, and uterus (for examples, see Fig. 1c). Unexpectedly, male-only expression was seen in the trachea, thyroid and parathyroid. No LacZ staining could be detected in the gut, brown or white adipose, pancreas, thymus, spleen, cartilage, bone, skeletal muscle, lung, mammary gland or peripheral nervous system; see Supplementary Table S1 for full list of tissues assessed and more expression data are available at www.mousephenotype.org.

**Fig. 2:**
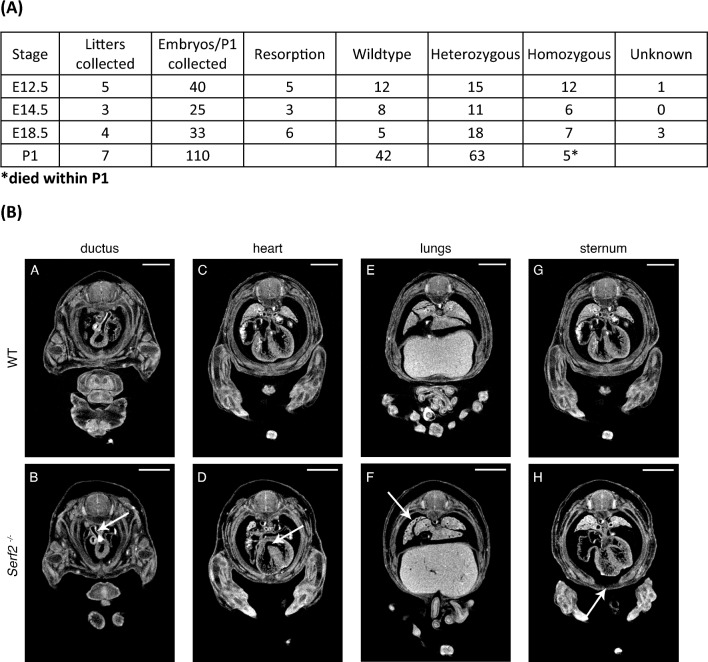
*Serf2* null knockout mice have developmental defects at E14.5 and are embryonic lethal on a C57BL6/Ntac background. **a** Table showing genotypes of E12.5, E14.5, E18.5-day embryos produced by a heterozygous *Serf2*^+/−^ intercross on the C57BL/6NTac background. **b** Micro CT 3D assessment of E14.5 wildtype (WT) and homozygous *Serf2*^*−/−*^ embryos, white arrows indicate site of defect: **A, B** altered angle of entry of the pulmonary trunk ductus arteriosis, see white arrow in **B**; **C, D** an interventricular septal defect, see white arrow in **D; E, F** delay in lung lobe development; **G, H** delay in rib fusion: sternum primordium, see white arrow in H. Scale bar = 1 mm

In this programme, embryonic lethality is defined by the collection of 28 embryos and none having the genotype of interest, at a particular timepoint. When pups were assessed from a heterozygous intercross, by this definition complete knockout of the *Serf2* gene (homozygous *Serf2*^*tm1b*^ allele) was lethal when mice were maintained on the C57BL/6Ntac background: when E18.5 embryos were sampled, genotype percentages were similar for wildtype, heterozygous and homozygous embryos (Fig. 2a). However, at birth, although we were able to genotype 5 homozygous P1 pups out of 110 total neonates, all 5 died within P1. This indicates substantial loss of homozygotes between E18.5 and P1.

Furthermore, from this intercross we found 42 wildtype mice at P1, and so would expect ~ 84 heterozygotes, whereas we found only 63 heterozygotes, showing loss of fitness for this genotype at this stage. We note we found no homozygous animals at weaning (Fig. 2a).

MicroCT analysis of six *Serf2* null compared to six wildtype littermate E14.5 embryos showed widespread developmental defects and reduced embryo size (embryo volume) (Fig. 2b). Defects include abnormal entry point/angle of the ductus into the heart in 3/6 animals, oedema (4/6), pulmonary trunk deficits (3/6), lung deficits (5/6) interventricular septal defects (4/6) and abnormal rib fusion (6/6). These data show incomplete penetrance and variable expressivity of dysmorphology defects of the *Serf2* null phenotype on the inbred C57BL/6Ntac background, in keeping with previous observations of other knockout animals on this background (Dickinson et al. 2016). Some of these deficits—the interventricular septal defect, lung lobe deficit and abnormal rib fusion, appear to resolve and are not present by E18.5 indicating developmental delay in the embryos (data available but not shown).

### *Serf2*^+/–^ heterozygous knockout male, but not female, mice have a deficit in startle response and pre-pulse inhibition

Adult heterozygous *Serf2* knockout mice (*Serf2*^*tm1b*^ allele) on the C57BL/6NTac background were put through a pipeline of standardised tests used by the International Mouse Phenotyping Consortium (IMPC) (data not shown, available at https://www.mousephenotype.org/data/genes/MGI:1337041).

**Fig. 3:**
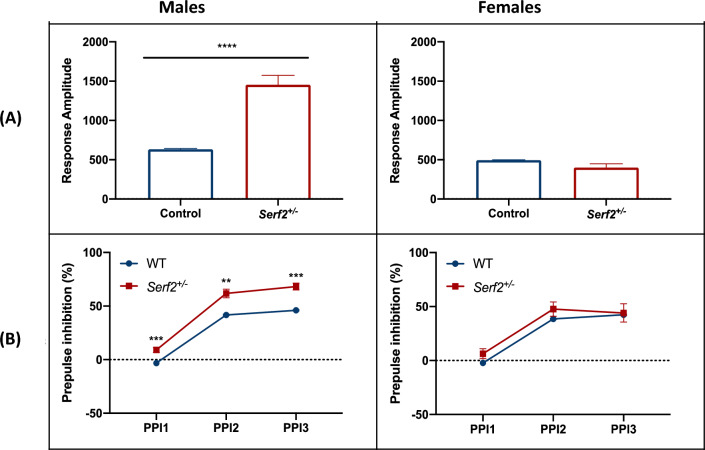
Acoustic startle and pre-pulse inhibition tests on wildtype and *Serf2* heterozygous knockout adults (C57BL/6NTac background). **a** Acoustic startle test. Acoustic startle testing was carried out with 1082 wildtype male, 1083 wildtype female, 8 *Serf2*^+/−^ males, and 7 *Serf2*^+/−^ females at 10 weeks of age. Animals were habituated to the chamber with a background noise of 53 dB for 5 min. ASR without pre-pulse. *Serf2*^+/−^ male mice exhibited significantly greater startle responses compared to WT mice (Mann–Whitney; Males: p < 0.0001, U = 367; Females: p = 0.2215, U = 2774). **b** Pre-pulse inhibition test. Pre-pulse inhibition (PPI) tests were carried out with 1082 wildtype male, 1083 wildtype female, 8 *Serf2*^+/−^ male, and 7 *Serf2*^+/−^ female mice at 10 weeks. Animals were habituated to the chamber with a background noise of 53 dB for 5 min. PPI at three different pre-pulse intensities (56, 58, 65 dB). PPI values were measured after the startle tone (120 dB), following a pre-pulse and an 80 ms gap at background noise. Ninety trials were performed in pseudorandom order, with the interstimulus interval varying from 20-30 s. *Serf2*^+/−^ male mice displayed significantly more PPI than wildtype mice at PPI1, PPI2, and PPI3 (unpaired t-test with Welch’s correction; PPI1: p = 0.0007, t = 5.323, df = 8.017; PPI2: p = 0.0014, t = 4.967, df = 7.303; PPI3: p = 0.0002, t = 6.863, df = 7.410)

At 10 weeks of age, *Serf2*^+/–^ male but not female mice had significantly increased response amplitudes in the acoustic startle test (Fig. 3a) and increased pre-pulse inhibition (Fig. 3b). No other significant differences from wildtype were detected in the standard testing data.

### *Serf2*^*−/−*^ null animals on a mixed genetic background survive to adulthood

When *Serf2*^+/−^ (*Serf2*^*tm1d*^ allele) heterozygous breeders, made congenic by maintaining on a C57BL/6 J background for 6 generations were subsequently crossed over two generations to a mixed 129S8, C57BL/6/Nimr genetic background (Cohort B), homozygous null *Serf2*^*−/−*^ progeny were produced that survived to adulthood i.e. at least 3 months of age. The percentages of pup genotypes on this mixed background were 15% wildtype, 65% heterozygous and 19% homozygous null littermates, indicating no significant loss of null animals (Chi Square test, p = 0.1). Note that these data are on the *Serf2*^*tm1d*^ allele, not the *Serf2*^*tm1b*^ allele as used above.

**Fig. 4:**
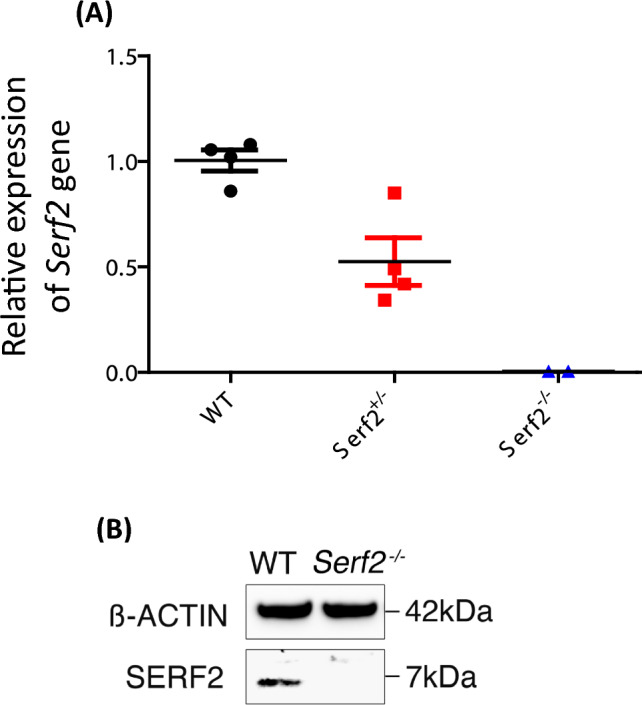
*Serf2* null mice are viable and do not express SERF2 on the mixed genetic background **a** Q-RT-PCR of cortex from 3-month-old wildtype, heterozygous and homozygous littermates shows no detectable expression of *Serf2* from the targeted null allele, *Serf2*^*tm1b*^ (black circles wildtype, red squares heterozygous, blue triangles homozygous littermates). **b** The 7 kDa SERF2 protein is not detectable in cortex of 3-month-old null animals

*Serf2* expression analysis on this hybrid background was performed by quantitative RT-PCR (qRT-PCR) on cortical homogenates from *Serf2*^*−/−*^ knockout, heterozygous *Serf2*^+/−^ and wildtype littermates at 3 months of age (Fig. 4a). Analysis showed that total expression of all *Serf2* isoforms was reduced by 50% in the heterozygous *Serf2*^+/−^ mice and was undetectable in the *Serf2*^*−/−*^ knockout mice, compared to expression levels in wildtype littermates. Loss of the 7 kDa SERF2 protein was confirmed by western blot analysis of cortical homogenates from null mice using wildtype littermates as controls (Fig. 4b).

Both the *Serf2*^*−/−*^ null and heterozygous *Serf2*^+/−^ animals (*Serf2*^*tm1d*^ allele) on the hybrid background, males and females, displayed general good health and lived beyond 3 months of age. There were no obvious differences between wildtypes, heterozygotes and homozygotes of both sexes. Phenotyping was not performed on these mice.

## Discussion

The SERF2 protein is highly conserved and has already been shown to play a role in protein binding and aggregation formation in *C. elegans* (van Ham et al. 2010) and *S. cerevisiae* models (Meinen et al. 2019), as well as human cell lines (Balasubramaniam et al. 2018). Our interest in this protein arose from our research into different forms of neurodegeneration and the need for an in vivo model to investigate the potential physiological and pathological roles of SERF2 in a whole mammalian organism. In this study we bred heterozygous and homozygous knockout animals on different genetic backgrounds in order to conduct standardised phenotyping using the IMPC pipeline. Our results show a modulating effect on SERF2 loss by unknown genes in the mouse genome, although we note the *Serf2*^*tm1d*^ allele survived on the mixed background rather than the *Serf2*^*tm1b*^ allele which was used on the inbred C57BL/6 N background; an effect of the individual knockout allele on embryonic lethality seems unlikely but could be investigated further in new mouse crosses. The knockout alleles indicate a role for SERF2 in the nervous system with significant behavioural and neurological deficits in mice with knockout alleles (Fig. 3), and also a wider role for this protein in development (Fig. 2) and in the adult (Fig. 1).

On the C57BL/6 N background *Serf2* heterozygous animals survived to adulthood but the null animals did not survive beyond P1 (Fig. 2, Table S2A). MicroCT imaging indicated developmental delay in homozygous null animals but this appeared to largely resolve towards term. Nevertheless, no live null mice were born on this genetic background.

Through analysing the LacZ expression pattern of the *Serf2*^+/–^ mouse with the tm1b allele (C57BL/6 N), we found expression was not ubiquitous and in part had a sex-specific pattern with expression in some male tissues but not the female equivalent (Fig. 1, Supplementary Table S1). At the transcript level, we note the widespread *SERF2* mRNA expression in the mouse and human nervous system, and in endothelial cells (www.brainrnaseq.org).

In heterozygous mice on the C57BL/6 N background we found deficits in acoustic startle response and in pre-pulse inhibition in adult males, not females, indicating some form of neuronal deficit in these animals (Fig. 3). The startle amplitude is higher in males than females (which are comparable to wildtype littermates) i.e. males jump further in response to the tone. The apparently increased pre-pulse inhibition may in part arise from the increased startle, but this response should be investigated further.

The male-specificity of gene expression in a few tissues and these behavioural phenotypes was an unexpected result from this autosomal gene, which should be investigated further in a larger cohort of mice.

On the mixed genetic background, we found homozygous null animals were produced at Mendelian ratios. We saw no obvious deficits in these mice, but it would be interesting to look using the standardised IMPC phenotyping pipeline and to behaviourally test these mice in depth, which we did not do.

Although SERF1 and SERF2 proteins share structural homology and have a similar biological function in vitro*,* interestingly the *Serf1* knockout mouse presents quite different phenotypes from the *Serf2* knockout mouse. These include increased circulating cholesterol levels and decreased bone mineral density in females, and increased heart weight in males (data available at https://www.mousephenotype.org/data/genes/MGI:1337114). These data suggest that, in vivo, SERF1 may have a role in cellular protein homeostasis.

Although we do not have expression data available for *Serf1,* qualitative *Serf2* adult expression data for the C57BL/6 N genetic background can be found at:

https://www.mousephenotype.org/data/genes/MGI:1337041#phenotypesTab

The *Serf2* locus is located on mouse chromosome 2, which has homology to human chromosome 15q15.3. Several schizophrenia susceptibility loci have been identified on chromosome 15 (Tsuang et al. 2001; Stephens et al. 2012; Wang, Liu, and Aragam 2010), including an auditory-evoked response inhibition phenotype associated with region 15q14 (Freedman et al. 1997). In addition, there is evidence for genetic linkage of chromosome 15 and bipolar disorder (Turecki et al. 2001; Vazza et al. 2007). A human deafness-infertility syndrome has also been mapped to this region, although likely due to a deletion ~ 100 kb away (Zhang et al. 2007). Intriguingly we find male-specific behavioural and neuronal phenotypes in our mice, given the evidence supporting the contribution of multiple 15q genes to schizophrenia (Stephens et al. 2012), this could be of significance. Interestingly the *SERF2* gene in both human and mouse is adjacent to, and in the same orientation as, huntingtin interacting protein K (*HYPK*). HYPK can act as a chaperone to suppress mutant huntingtin aggregates (Raychaudhuri et al. 2008). Connected via a conjoined gene these two genes are regulated by a common transcription factor yet their protein products have opposite cellular functions in regulating protein aggregation (Das and Bhattacharyya 2016).

Given the known role of SERF proteins in modulating aggregation, these *Serf2* KO mice provide an in vivo platform to investigate the role of SERF2 protein in the pathology of neurodegenerative diseases. Crossing the mice to mouse models of neurodegeneration will provide new insights into disease-related protein aggregation and potentially advance the identification of new therapeutic targets. Finally, all *Serf2* knockout alleles described here, and the conditional allele *Serf2*^*tm1c*^, are freely available via the International Mouse Knockout Project at https://www.mousephenotype.org

## Supplementary Information

Below is the link to the electronic supplementary material.

Supplementary file1 (docx 18 kb)

## Data Availability

All phenotyping data as mentioned in the text is available through the International Mouse Phenotyping Consortium (IMPC) website www.mousephenotype.org). As stated, all *Serf2* knockout mice mentioned in the text are available through IMPC.
