# Supplementary material for: A novel knockout mouse for the small EDRK-rich factor 2 (Serf2) showing developmental and other deficits
Source: Mamm Genome. 2021 Mar 13;32(2):94–103. doi: 10.1007/s00335-021-09864-6 (PMC8012326; doi:10.1007/s00335-021-09864-6)
Supplement: Supplementary file 1 — Supplementary file1 (docx 18 kb) [file 335_2021_9864_MOESM1_ESM.docx]

**Supplementary Table 1. Adult mouse tissues,** *Serf2^tm1b^* **heterozygous animals, assessed by X gal staining for LacZ expression from the *Serf2* locus.**

| **Animal** | ***Serf2* ^+^/^-^ Female** | ***Serf2* ^+^/^-^ Male** |
| --- | --- | --- |
| Brown Adipose tissue | A | A |
| White Adipose Tissue | A | A |
| Heart | PRESENT | PRESENT |
| Blood Vessel | PRESENT | PRESENT |
| Aorta | A | A |
| Stomach | A | A |
| Small Intestine | A | A |
| Large Intestine | A | A |
| Liver | A | A |
| Oesophagus | A | A |
| Gall Bladder | A | A |
| Oral Epithelium | PRESENT | PRESENT |
| Adrenal Gland | PRESENT | PRESENT |
| Thyroid Gland | A | PRESENT |
| Parathyroid Gland | A | PRESENT |
| Pancreas | A | A |
| Thymus | A | A |
| Spleen | A | A |
| Peyer's Patch | A | A |
| Mesenteric lymph node | A | A |
| Skin | A | A |
| Cartilage | A | A |
| Skeletal Muscle tissue | A | A |
| Bone | A | A |
| Olfactory Bulb | PRESENT | PRESENT |
| Cerebral Cortex | PRESENT | PRESENT |
| Striatum | PRESENT | PRESENT |
| Hippocampus | PRESENT | PRESENT |
| Hypothalamus | PRESENT | PRESENT |
| Cerebellum | PRESENT | PRESENT |
| Brainstem | PRESENT | PRESENT |
| Brain | PRESENT | PRESENT |
| Spinal cord | PRESENT | PRESENT |
| Peripheral Nervous System | A | A |
| Pituitary Gland | PRESENT | PRESENT |
| Kidney | PRESENT | PRESENT |
| Lower Urinary Tract | PRESENT | PRESENT |
| Mammary gland | A | A |
| Ovary | PRESENT | - |
| Oviduct | PRESENT | - |
| Uterus | PRESENT | - |
| Testis | - | PRESENT |
| Prostate Gland | A | A |
| Trachea | A | PRESENT |
| Lung | A | A |
| Eye | A | A |

Male and female adult tissues were scored for presence or absence of β-galactosidase expression using X gal staining by independent experimenters. Lists were then compared and any ambiguity between sexes was then checked and confirmed; A means absence of expression. More expression data are available at www.mousephenotype.org.
